# Effects of a single dose of beetroot juice on cycling time trial performance at ventilatory thresholds intensity in male triathletes

**DOI:** 10.1186/s12970-018-0255-6

**Published:** 2018-10-04

**Authors:** Manuel Vicente Garnacho-Castaño, Guillem Palau-Salvà, Eduardo Cuenca, Arturo Muñoz-González, Pablo García-Fernández, María del Carmen Lozano-Estevan, Pablo Veiga-Herreros, José Luis Maté-Muñoz, Raúl Domínguez

**Affiliations:** 1 Research group in physical activity, performance and health (GRI-AFIRS), School of Health Sciences, TecnoCampus-Pompeu Fabra University, Ernest Lluch, 32 (Porta Laietana) 08302 Mataró, Barcelona, Spain; 2 Laboratory of Biomechanics and Exercise Physiology, Department of Physical Activity and Sports Science, Alfonso X El Sabio University, Avenida Universidad, 1, 28691 Villanueva de la Cañada, Madrid, Spain

**Keywords:** Cardioventilatory responses, Gross mechanical efficiency, Cycling efficiency, Slow component, Energy expenditure

## Abstract

**Background:**

Beetroot juice (BJ) is classified as a high-level supplement for improving sports performance. There is some controversy over the benefits of BJ supplementation for endurance exercise performance, especially when referring to well-trained athletes. This study examines the effects of acute BJ supplementation on cardioventilatory responses, exercise economy/efficiency, slow component of oxygen uptake, time trial performance, blood lactate, energy consumption, and carbohydrate and fat oxidation.

**Methods:**

Twelve well-trained, male triathletes (aged 21–47 yr) were assigned in a randomized, double-blind, crossover design to receive 70 ml of BJ (6.5 mmol NO_3_^−^) or placebo (PL). Three hours after taking the supplement, participants completed an endurance test on a cycle ergometer at a constant work rate (W) corresponding to first ventilatory threshold (VT1) (30 min) and second ventilatory threshold (VT2) time trial (~ 15 min).

**Results:**

Maximal oxygen uptake was 54.78 ± 3.13 mL·min^− 1^·kg^− 1^, and gross efficiency was > 22% at each load intensity and experimental condition. No significant interaction effect (supplement*intensity) was observed on any of the cardioventilatory variables, efficiency/economy, VT2 time trial, energy expenditure, carbohydrate oxidation and fat oxidation (*p* > 0.05).

**Conclusion:**

Our findings do not support an improvement in the variables examined in response to acute BJ supplementation. Probably, higher doses are needed for improving time trial performance in male triathletes during a cycle ergometer test conducted at a load intensity equivalent to the first and second ventilatory threshold.

## Background

Beetroot juice (BJ) is classified as a supplement of high scientific evidence for improving sports performance [1]. It is characterized by its high nitrate content (NO_3_^−^) which, after ingestion, is actively extracted and concentrated in the saliva. Here NO_3_^−^ is reduced to nitrite (NO_2_^−^) by bacteria in the mouth. In turn, NO_2_^−^ may be further reduced in the stomach and muscle to nitric oxide (NO) [2, 3]. NO is an important signaling molecule with a key role in several physiological processes which may affect exercise performance such as regulating tissue blood flow, muscle contraction, respiration and mitochondrial biogenesis, and muscle glucose uptake [4].

In animal studies, it has been demonstrated that NO_3_^−^ supplementation elevates skeletal muscle O_2_ delivery and improves vascular control during exercise predominantly in fast-twitch type II muscles. Furthermore, NO_3_^−^ supplementation improves metabolic control [5]. A human study has suggested that NO_3_^−^ supplementation may enhance physiological and functional responses in type II muscle fibers [6]. These potential physiological mechanisms induced by NO_3_^−^ supplementation on type II muscle fibers could justify, at least in part, improvements in performance during intense exercise in healthy adults, and for improving functional capacity in senescent and patient populations [6].

It has been well established that performance at endurance exercise is linked to maximum oxygen uptake (VO_2max_), lactate threshold, ventilatory threshold (VT), exercise economy/efficiency [7–11] and VO_2_ kinetics [12]. Acute and chronic supplementation with NO_3_^−^ has been shown to reduce O_2_ cost in various forms of exercise, with different supplementation protocols and at different exercise intensities. NO_3_^−^ reduced O_2_ cost during knee-extensor exercise (6 days, 0.5 l/day NO_3_^−^, 5.1 mmol/day) [13], decreased during high intensity exercise(6 days, 0.5 l/day NO_3_^−^, 5.5 mmol/day) [14] and submaximal exercise (1 day and 15 days, 0.5 l/day, 5.2 mmol/day of NO_3_^−^) [15] in cycle ergometer, and diminished during walking and moderate- and severe-intensity running (6 days, 0.5 l/day NO_3_^−^, ~ 6.2 mmol/day) [16]. In addition, NO_3_^−^ supplementation improved muscle contractile efficiency, increased time to exhaustion by 25%, reduced the amplitude of the VO_2_ slow component by 50% [13], and diminished cycle time trial in trained (6 days, 140 ml/day NO_3_^−^, ~ 8 mmol/day) [17] and competitive cyclists (1 day, 0.5 l NO_3_^−^, ~ 6.2 mmol) [18]. Because of these findings, it has been proposed that BJ supplementation could have an ergogenic effect in athletes [1] especially when executing long-duration, endurance exercise modalities [19].

There is some controversy over the benefits of BJ supplementation for endurance exercise performance, mainly when referring to highly-trained athletes. In a study performed in elite cyclists, it was found that BJ (6 days, 0.5 l/day NO_3_^−^, ~ 0.5 g/day, 820 KJ per drink) failed to improve performance, exercise economy and VO_2_ kinetics measured in a 2 h preload test and a 400 kcal time trial [20]. Cermak et al. (2012) [21] also observed that acute BJ intake (500 ml, ~ 6.2 mmol NO_3_^−^) did not improve power, time-trial performance or heart rate response in a 1 h cycle time trial in trained cyclists.

International Olympic Committee (IOC) consensus statement [22] determines that improvements in performance after acute BJ supplementation are commonly detected within 2–3 h following a NO_3_^−^ bolus of 5–9 mmol (310–560 mg) [23]. Longer periods (> 3 days) of NO_3_^−^ supplementation appears to increase sport performance [24, 25], especially, when performance gains appear harder to achieve in highly-trained athletes [12]. Higher doses of NO_3_^−^ (> 8 mmol) have shown to improve performance in trained rowers [23]. There are some uncertainties for the dose-response relationship exists between biological mechanisms and acute BJ supplementation for improving endurance performance in well-trained athletes. The differences observed between highly-trained competitive athletes and amateur athletes in the effects caused by BJ supplementation could be a consequence of years of training adaptations and genetic factors [26].

The physiological mechanisms underlying the impacts of NO_3_^−^ supplementation on cardiorespiratory endurance performance remain unclear. Studies have shown that factors such as NO_3_^−^ dose, training level, athlete status, duration of supplementation (acute or chronic), regular dietary NO_3_^−^ intake and exercise test duration and intensity may all affect the impacts of BJ consumption [12]. It remains clear that much further work is needed to elucidate the physiological adaptations and responses induced by BJ in trained or even untrained subjects before and after a training intervention [20].

In the studies performed to date, different test protocols have been used to assess the impacts of NO_3_^−^ supplementation and there is some debate as to which tests are the most suitable for assessing endurance performance. Tests of time to exhaustion measure exercise capacity more than performance per se. These protocols have been criticized for their deficient ecological validity and their limited applicability to some sports modalities [27, 28]. Other studies have based their assessments on covering a given distance in the fastest time possible (time trial) as an intervention to improve sport performance [27, 29].

The arduous nature of laboratory cycling time trials means it is not possible to ask participants to execute a familiarization trial at the criterion distance, which could indicate a lack of knowledge of the performance variability of cyclists in time trials, especially in subjects not experienced in cycling [30]. Given the complexity of designing specific tests to simulate real sports conditions (lab tests vs races), we propose opting for a test conducted at an intensity equivalent to VT, in which aerobic performance (first ventilatory threshold or VT1) and transition towards an anaerobic energy metabolism (second ventilatory threshold or VT2) can be assessed. The use of submaximal VT workloads seems to more accurately predict cycling endurance performance [31]. This has been described as a valid method [32] that shows a direct relationship between VT and 40 km time trial performance. Moreover, the gas exchange threshold and VT are highly correlated with running velocity in triathlon and marathon tests [33–35], and VT2 is a strong predictor of performance in time-trials [36].

The objective of the present study was to assess the effects of acute BJ supplementation on endurance exercise performance and cardioventilatory responses in well-trained triathletes during a cycle ergometer test conducted at a load intensity equivalent to the first and second ventilatory threshold. Our working hypothesis was that a single dose of BJ supplementation would improve cardiorespiratory endurance performance by diminishing $\dot{\mathrm{V}}$O_2_ for a given workload by means of more efficient and economic mechanical and energy-producing physiological mechanisms.

## Methods

### Participants

Participants recruited were 12 well-trained triathletes at the national (*N* = 8) and international (*N* = 4) level (age, 39.3 ± 7.5 years; height, 176.5 ± 7.5 cm; weight, 72.8 ± 6.9 kg; BMI, 23.4 ± 2.2; VO_2_max, 54.8 ± 3.1 mL·min^− 1^·Kg^− 1^) from different triathlon clubs in Madrid (Table 1). Participation was voluntary though we established the following inclusion criteria: a) national and/or international competition level; b) VO_2max_ > 50 mL·min^− 1^·kg^− 1^ in cycling; c) no cardiovascular, respiratory, metabolic, neurological or orthopedic disorders that could affect cycle ergometer performance; d) no consumption of drugs or medication; e) no smoking; and f) no nutritional supplements taken in the three months before the study outset (e.g., caffeine, β-alanine, creatinine, sodium bicarbonate, glutamine, etc.). To be classified as well-trained, subjects had to have undergone training for at least 1 h at least 4 times per week and have competed in at least one organized cycle race in the preceding 12 months [30]. Sample size calculation was based on the results of a pilot study with the same study protocol involving 10 sport science students. The calculation of sample size was performed with α = 0.05 (5% chance of type I error) and 1- β = 0.80 (power 80%), and applying the results provided from previous studies, which used the same [17] or a smaller sample size [18]. A total of 12 well-trained triathletes was required for this study to detect differences between both experimental conditions.

**Table 1:** Descriptive characteristics of national and international triathletes

	National Level	International Level
Participants	*n* = 8	*n* = 4
Age (years)	39.4 (8.1)	39.0 (7.6)
Height (cm)	175.0 (7.3)	179.5 (8.1)
Weight (kg)	72.7 (5.1)	73.1 (10.8)
BMI	23.8 (2.4)	22.5 (1.6)
VO_2_max (mL.kg^− 1^.min^− 1^)	53.1 (2.1)	58.3 (1.7)

Data are provided as mean ± standard deviation (SD) Abbreviations used: *BMI* body mass index, *VO*_*2max*_ maximum oxygen uptake

The subjects were informed of the study goals and test protocols before giving their signed informed consent for participation. The study protocol received approval from the Ethics Committee of the Universidad Alfonso X El Sabio (Madrid, Spain).

### Study design

Participants completed three cycle ergometer test sessions at our Exercise Physiology laboratory. As in previous studies [37, 38], a washout period of at least 72 h separated the laboratory visits. Sessions were conducted under the same ambient conditions (temperature 20 °C–22.5 °C, relative humidity 42–52%) and in the same time frame (+ 0.5 h). Participants refrained from any high-intensity physical effort from 72 h and refrained from any type of physical exercise from 24 h before starting the first session to the study end. They were allowed to perform low intensity workouts, except 24 h before the start of the test.

In Session 1, an incremental test until exhaustion was performed on a cycle ergometer. In this test, determination was made of maximum or peak cardioventilatory indices and ventilatory thresholds VT1 and VT2. The power output (in W) eliciting VT1 and VT2 was recorded to determine the workload for the constant test at the intensity of VT1 and VT2 during sessions 2 and 3.

Sessions 2 and 3 were identical and both experimental conditions were compared BJ vs. placebo (PL). In these sessions, supplement assignment was double blind fashion and random. Participants took the supplement given to them, BJ or PL, as soon as they arrived at the lab ensuring that 50% of the triathletes randomly took PL in the first session and BJ in the second or vice versa. This meant that half the participants in each session worked under one of the two experimental conditions. Three hours after taking the supplement, the athletes started with a warm up before conducting an endurance test on a cycle ergometer at a constant workload (W) corresponding to VT1 (30 min) and, without rest, at a constant workload set at VT2 intensity (VT2 time trial) (~ 15 min). After VT2 time trial, participants answered a few questions to verify whether they were blinded to the supplementation condition. During 3-h period post BJ ingestion and before beginning the test, the triathletes remained under resting conditions.

### Diet and supplementation

As an individual’s diet can affect energy metabolism during exercise [39], subjects were given guidelines by a qualified nutrition professional to ensure that 48 h before each of the test sessions, they followed a similar diet consisting of ~ 60% carbohydrates (5.5 g carbohydrate per kg), 30% lipids and 10% proteins. Dietary ingestion was controlled during the 48-h period before each test session by means of a combination of usual diet and the nutritionist’s recommendations. The diet consisted of typical food sources recommended for endurance athletes (e.g., bread, pasta, rice, milk, chicken, tuna, fruit, etc.) considering the energy intake from the PL and BR beverages.

The triathletes recorded their diet for the 48-h period before the first experimental test and replicated the same diet during the 48 h before the second trial. Upon arrival at the laboratory on a test day, participants’ diaries were evaluated by a nutrition expert to determine compliance with established dietary instructions. In the case of not complying with the guidelines, the athlete was excluded from the study.

They were also provided with a list of foods with high NO_3_^−^ contents they should avoid at least two days before the study outset (beetroot, celery, ruccula, lettuce, spinach, turnip, endives, leak, parsley, cabbage). Subjects were also instructed to avoid drinks containing caffeine or alcohol during the 24 h before the tests. No caffeine or alcohol intake was allowed during the study for avoiding any interaction with BJ.

Supplementation was given 3 h before the start of each test [19], as it has been established that NO_2_^−^ peaks in blood 2–3 h after the intake of NO_3_^−^ [40]. Each subject took the supplement by drinking the contents of a randomly assigned bottle containing 70 ml (~ 6.5 mmol, 404 mg of NO_3_^−^) of BJ concentrate Beet-It-Pro Elite Shot (Beet IT; James White Drinks Ltd., Ipswich, UK) or PL. The PL was a nitrate-depleted source and was prepared by dissolving 1 g of powdered BJ (~ 0.005 mmol, 0.311 mg of NO_3_^−^, ECO Saludviva, Alicante, Spain) in a litre of mineral water and adding lemon juice to imitate the taste of the commercial supplement. The PL supplementation was prepared by experts in nutrition and dietetics, and pharmacy. Both drinks (BJ and PL) were supplied in an unlabeled, 100-ml, brown glass bottle. During this period before the start of each test, the triathletes did not ingest food and fluids, apart from water, to guarantee hydration status.

Participants were asked to refrain from brushing their teeth or using a mouthwash, chewing gum or sweets that could contain a bactericidal substance such as chlorhexidine or xylitol in the 24 h prior to the test sessions. The reason for this is that the use of oral antiseptics can prevent increased blood NO_2_^−^ levels after the intake of NO_3_^−^ due to their effects on mouth bacteria [41].

All participants were warned of the possible side-effects of BJ, ie, gastrointestinal symptoms and the red appearance of urine and feces.

### Cycle ergometer tests

We used an Ergoselect 200 cycle ergometer (Ergoline GmbH, Bitz, Germany) for the incremental and submaximal tests, that was calibrated and adjusted for use with the corresponding pedals and participants’ footwear.

To measure the ventilatory variables, we used a gas analysis system (Ergostik, Geratherm Respiratory, Badd Kissingen, Germany) which was calibrated before each test using known O_2_ and CO_2_ concentrations and low, medium and high flow to calibrate ventilation. Gas exchange data were taken breath-by-breath to obtain the variables VO_2max_, minute ventilation (VE), ventilatory equivalent for oxygen (VE·VO_2_^− 1^), ventilatory equivalent for carbon dioxide (VE·VCO_2_^− 1^), respiratory exchange ratio (RER), end-tidal partial pressure of oxygen and carbon dioxide (PetO_2_ and PetCO_2_ respectively). Heart rate was measured by telemetric recording using a transmitter fixed to the chest that sent data to a portable receiver (RS-800CX, Polar Electro OY; Kempele, Finland). Ventilatory and heart rate data were transferred to a PC for subsequent analysis.

Warm-up consisted of 5 min cycling at a light rhythm for the incremental test (Session 1), with subjects selecting the workload and cadence. Next, the triathletes started a ramp test until exhaustion with an initial 50 W load that was gradually increased in 25 W per minute (5 W every 12 s). The participants cycled at a self-selected pedal rate of between 70 to 90 rpm. The test was voluntarily terminated by the athletes when cadence dropped to below 70 rpm, or at the point of extenuation.

The VO_2max_ was taken as the highest 30-s mean value attained prior to exhaustion in the test [16]. After the test, the criteria used to determine VO_2max_ were [42]: (1) a plateau produced in the VO_2_ curve with increases lower than 1.5 mL · kg^− 1^ · min^− 1^ between 30 s intervals; (2) RER above 1.10; and (3) a heart rate equal to or greater than the theoretical maximum. Maximum heart rate was recorded as the highest value obtained in the incremental test.

In addition to VO_2max_, two investigators separately identified VT1 and VT2. If there was lack of agreement, the opinion of a third observer was sought. We defined VT1 as the workload at which increases were produced in both VE∙VO_2_^− 1^ and PetO_2_, without a concomitant increase in VE∙VCO_2_^− 1^. Similarly, VT2 was determined when increases were produced in VE∙VO_2_^− 1^ and VE∙VCO_2_^− 1^, but this time accompanied by a drop in PetCO_2_ [43, 44].

Sessions 2 and 3 were preceded by the same warm-up as for the incremental load test. The ensuing test protocol consisted of 30 min of pedaling at a freely selected rate between 70 and 90 rpm at a constant workload equivalent to VT1, plus a VT2 time trial (~ 15 min), to try to complete the whole test time of ~ 45 min. Ventilatory data were recorded as means at 30 s time intervals. The workload (in W) was selected for each individual from the VT1 and VT2 values determined in the incremental test.

The slow component of the exercise test was defined as the difference (Δ VO2, in mL ^.^ min^− 1^) between VO_2_ at the end of exercise and VO_2_ at the end of the third minute of exercise at a constant load, both at VT1 and VT2. The values for the end of minute 3 were taken as the mean of VO_2_ from 2 min 40 s to 3 min 20 s, while those recorded at the end of exercise were the mean of the VO_2_ values obtained for the last 2 min [45].

Mean cycling efficiency (CE) at VT1 and VT2 were expressed in W · L^− 1^ · min^− 1^ while gross mechanical efficiency (GE) was calculated as the ratio of work accomplished per minute (ie, W in kcal · min^− 1^) to energy consumed per minute (ie, in kcal · min^− 1^), as described elsewhere [46]. Energy expenditure was calculated from VO_2_ and the RER using the tables of Lusk **(**VO_2_ L. min^− 1^ · RER expressed in kcal ^.^ L^− 1^ O_2_) [47].

The following equations were used to calculate the rates of carbohydrate and fat oxidation [48]:

$$ \mathrm{Carbohydrate}\ \mathrm{oxidation}\ \left(\mathrm{g}\cdotp {\min}^{-1}\right)=4.585\cdotp \left({\mathrm{VCO}}_2\right)-3.226\cdotp \left({\mathrm{VO}}_2\right) $$

$$ \mathrm{Fat}\ \mathrm{oxidation}\ \left(\mathrm{g}\cdotp {\min}^{-1}\right)=1.695\cdotp \left({\mathrm{VO}}_2\right)-1.701\cdotp \left({\mathrm{VCO}}_2\right). $$

Blood lactate concentrations were measured in each participant by an experienced investigator using the analyzer Lactate ProTM 2 (Arkray Factory Inc., KDK Corporation, Shiga, Japan). Clean blood samples (5 μl) were obtained from the index finger of the left hand. Lactate measurements were made: 1) at rest, 2) 30 s before the end of the VT1 stage, and 3) at the end of the test (VT2).

Participants graded their fatigue using the subjective rating of perceived effort [49] at the same time points as the lactate determinations.

### Statistical analysis

The Shapiro-Wilk test was used to check the normal distribution of the data, which are reported as mean and standard deviation (SD), mean and confidence intervals (95% CI) or percentage (%). A two-way ANOVA with repeated measures, supplement * intensity (BJ, PL * VT1, VT2, VT1 + VT2), was used to compare the effects of the two experimental conditions (BJ vs. PL) on the cardioventilatory, economy/efficiency, and metabolic variables during the constant-load test conducted at the intensity of VT1 and VT2. When appropriate, Greenhouse-Geisser probability levels were used to adjust for sphericity and Bonferroni adjustments were used to control for multiple post-hoc comparisons. A Student t-test for paired data was used to determine differences between BJ and PL. To determine the magnitude of the response to both experimental conditions (supplements) we estimated partial eta-squared (η_p_^2^). The scale for classification of η_p_^2^ was 0.01 = small, 0.06 = medium, 0.14 = large. We also calculated the probability of demonstrating the effectiveness of each supplement through statistical power (SP). Significance was set at *p* < 0.05. All statistical tests were performed using the software package SPSS version 19.0 for Macintosh (SPSS Inc., Chicago, IL, USA).

## Results

Intake of BJ and PL supplementation was well tolerated by all participants of the study, however, some triathletes showed beeturia (red urine) and red stools. Participants ingested the prescribed dose of BJ and PL as determined by the nutritionist and their dietary interventions were consistent with established dietary guidelines. After the completion of the tests, all subjects were unable to differentiate between BJ and PL condition and, therefore, the triathletes were blinded to the supplementation condition.

### Cardioventilatory responses and VO_2_ kinetics

The cardioventilatory variables measured in the incremental test until exhaustion (Session 1) are shown in Table 2 and those recorded at VT1 and VT2 in the constant load tests are provided in Table 3.

**Table 2:** Cardioventilatory parameters and load obtained in incremental test

Variable	VT1	VT2	VO_2max_
Power (W)	195.4 (43.3)	282.1 (37.9)	390.3 (52.8)
VO_2_ (L·min^− 1^)	2.2 (0.4)	3.0 (0.3)	3.9 (0.5)
VO_2_·Kg^− 1^ (mL·min^− 1^·Kg^− 1^)	26.5 (11.9)	42.1 (4.6)	54.8 (3.1)
VCO_2_ (L·min^− 1^)	2.1 (0.5)	3.2 (0.4)	4.9 (0.8)
RER	0.9 (0.1)	1.1 (0.0)	1.3 (0.1)
V_E_ (L·min^− 1^)	56.3 (10.7)	87.7 (14.2)	167.7 (36.5)
V_E_·VO_2_^− 1^	24.7 (2.4)	28.1 (2.0)	41.2 (4.5)
V_E_·VCO_2_^−1^	26.8 (2.1)	26.8 (1.9)	32.3 (2.7)
PetO_2_ (mmHg)	90.8 (4.2)	95.3 (3.1)	106.6 (2.8)
PetCO_2_ (mmHg)	32.4 (2.9)	32.4 (2.6)	27.0 (2.4)
HR (beats·sec^−1^)	120.2 (13.5)	146.3 (13.1)	169.3 (12.8)
Intensity (% of VO_2max_)	55.8 (10.2)	75.7 (6.6)	–

Data are provided as mean ± standard deviation (SD) and percentage (%)

*Abbreviations: HR* heart rate, *PETCO*_*2*_ end-tidal partial pressure of carbon dioxide, *PETO*_*2*_ end-tidal partial pressure of oxygen, *RER* respiratory exchange ratio, *VCO*_*2*_ carbon dioxide, *VE* minute ventilation, *VE·VCO*_*2*_^*− 1*^ ventilatory equivalent for carbon dioxide, *VE·VO*_*2*_^*− 1*^ ventilatory equivalent for oxygen, *VO*_*2*_ oxygen uptake, *VO*_*2max*_ maximum oxygen uptake, *VT1* first ventilatory threshold, *VT2* second ventilatory threshold

**Table 3:** Comparison between beetroot juice (BJ) supplementation and placebo (PL) experimental condition on cardiorespiratory variables

	EC	VT1	VT2	Total (VT1 + VT2)	*P* ^*1*^	*P* ^*2*^
HR(beats·sec^−1^)	BJ	130.7 (17.3)	159.6 (11.7)	145.1 (13.8)	0.517	0.485
	PL	129.4 (17.2)	160.0 (14.0)	144.3 (14.0)		
VO_2_ (L·min^−1^)	BJ	2.4 (0.4)	3.4 (0.3)	2.9 (0.3)	0.241	0.493
	PL	2.4 (0.5)	3.3 (0.4)	2.9 (0.4)		
VO_2_ (%)	BJ	61.8 (11.3)	85.9 (7.6)	73.8 (8.9)	0.253	0.512
	PL	60.5 (12.2)	83.9 (8.3)	72.2 (9.4)		
VCO_2_ (L·min^−1^)	BJ	2.5 (0.4)	3.7 (0.4)	3.1 (0.3)	**0.001**	0.579
	PL	2.4 (0.5)	3.5 (0.5)	2.9 (0.4)		
RER	BJ	0.9 (0.0)	1.1 (0.1)	1.0 (0.0)	0.106	0.623
	PL	0.9 (0.1)	1.1 (0.1)	1.0 (0.0)		
V_E_ (L·min^−1^)	BJ	74.9 (14.6)	127.6 (19.9)	101.2 (15.9)	0.054	0.622
	PL	72.9 (16.8)	125.5 (23.5)	96.8 (16.8)		
V_E_·VO_2_^−1^	BJ	28.9 (2.1)	36.3 (4.5)	32.6 (2.9)	0.483	0.587
	PL	28.4 (1.9)	36.5 (5.7)	31.6 (2.5)		
V_E_·VCO_2_^−1^	BJ	28.9 (1.6)	33.4 (3.1)	31.1 (2.1)	0.162	0.573
	PL	29.3 (1.6)	34.2 (4.4)	31.1 (1.9)		

Data are provided as mean ± standard deviation (SD) and percentage (%)

*Abbreviations: EC* experimental condition, *HR* heart rate, *RER* respiratory exchange ratio, *VCO*_*2*_ carbon dioxide, *VE* minute ventilation, *VE·VCO*_*2*_^*−1*^ ventilatory equivalent for carbon dioxide, *VE·VO*_*2*_^*− 1*^ ventilatory equivalent for oxygen, *VO*_*2*_ oxygen uptake, *VO*_*2max*_ maximal oxygen uptake, *VT1* first ventilatory threshold, *VT2* second ventilatory threshold

^1^Significant differences for supplementation effect

^2^Significant differences for supplementation x intensity interaction

No significant interaction effect (supplement*intensity) was observed on any of the cardioventilatory variables (*p* > 0.05). The only significant effect found was that of the supplement (BJ, PL) on VCO_2_ (F_(1, 11)_ = 20.155, *p* = 0.001, η_p_^2^ = 0.647, SP = 0.983). No other effects of the supplement were noted (*p >* 0.05). Intensity effects were produced on heart rate (F_(2, 22)_ = 89.325, *p* < 0.001, η_p_^2^ = 0.890,SP = 1), VO_2_ (F_(2, 22)_ = 51.293, *p* < 0.001, η_p_^2^ = 0.823, SP = 1), %VO_2max_ (F_(2, 20)_ = 95.114, *p* < 0.001, η_p_^2^ = 0.905, SP = 1), VCO_2_ (F_(2, 22)_ = 56.529, *p* < 0.001, η_p_^2^ = 0.837, SP = 1), RER (F_(2, 22)_ = 29.670, *p* < 0.001, η_p_^2^ = 0.730, SP = 1), VE (F_(2, 22)_ = 127.248, *p* < 0.001, η_p_^2^ = 0.920, SP = 1), VE. VO_2_^− 1^ (F_(2, 22)_ = 36.048, *p* < 0.001, η_p_^2^ = 0.766, SP = 1), VE. VCO_2_^− 1^ (F_(2, 22)_ = 22.244, *p* < 0.001, η_p_^2^ = 0.669, SP = 1).

**Fig. 1:**
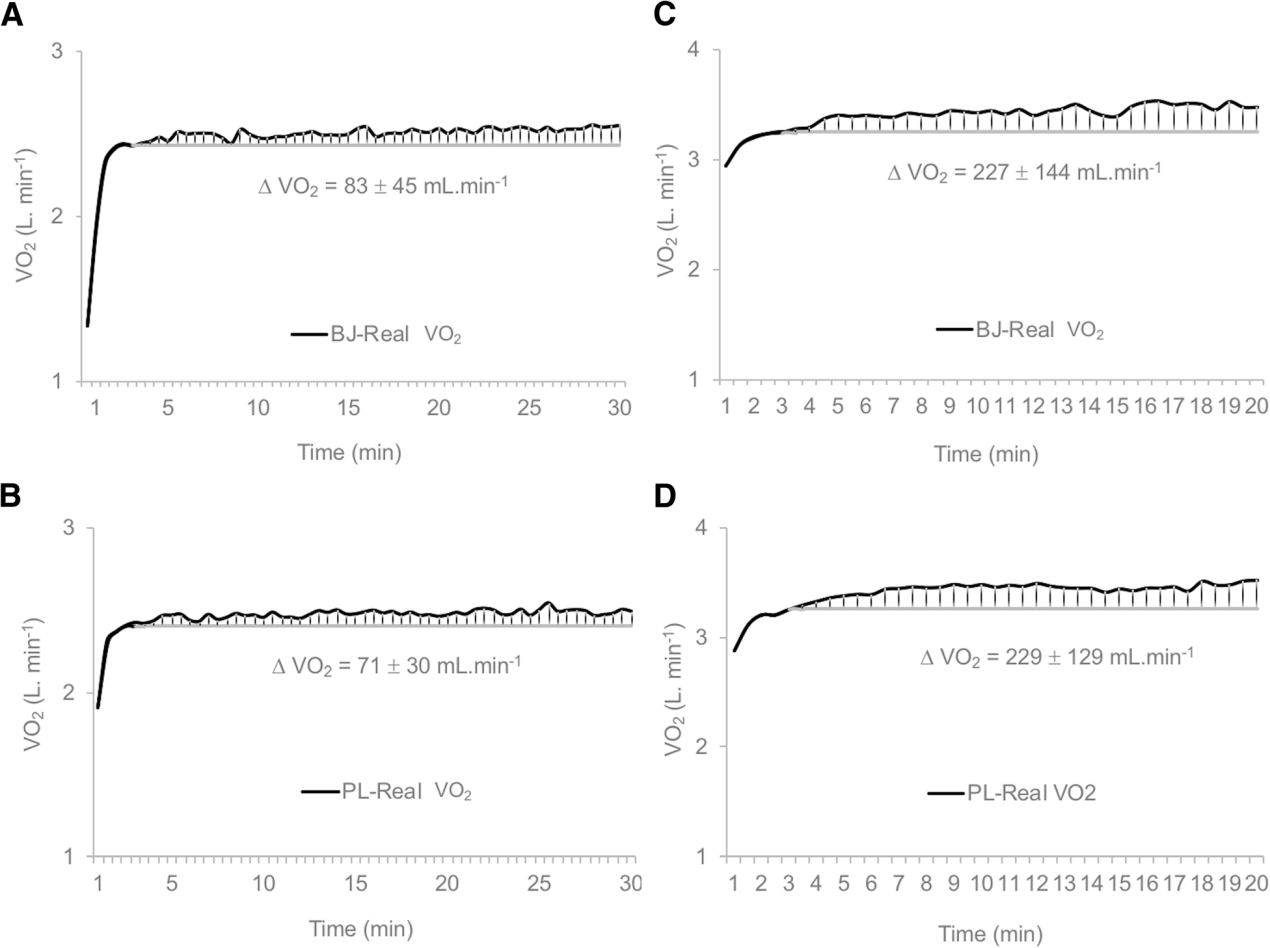
Slow component (ΔVO_2_ mL . min^− 1^) analysis during the constant load test in: **a** Beetroot juice (J) experimental condition at first ventilatory threshold (VT1); **b** Placebo (PL) experimental condition at VT1 **c** Beetroot juice (BJ) experimental condition at second ventilatory threshold (VT2); **d** PL experimental condition at VT2. Data are provided as mean and standard deviation. There were no significant differences between both experimental conditions (BJ vs. PL) in VT1 and VT2

No significant impacts of the supplements (BJ vs PL) (*p* > 0.05) were detected on VO_2_ kinetics measured through the slow component. A similar slow component was observed in both experimental conditions throughout the testing protocol in VT1 (BJ: 83 ± 45 mL. min^− 1^; PL: 71 ± 30 mL. min^− 1^) and in VT2 time trial (BJ: 227 ± 144 mL. min^− 1^; PL: 229 ± 129 mL. min^− 1^) (Fig. 1).

### Cycling efficiency, gross mechanical efficiency, lactate, VT2 time trial

**Fig. 2:**
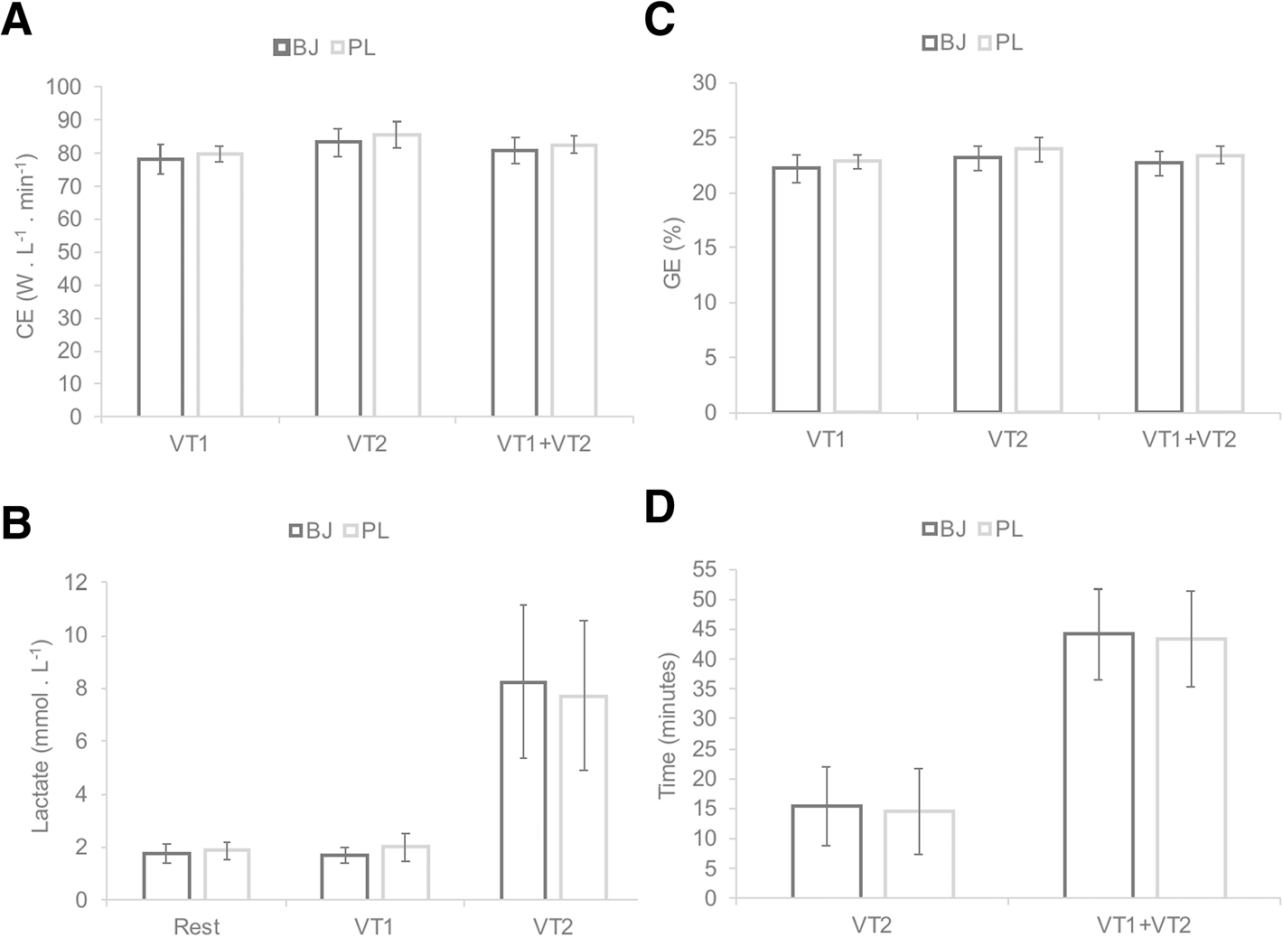
Differences between beetroot juice (BJ) and placebo (PL) at first ventilatory threshold (VT1), second ventilatory threshold (VT2) and in the total time of the test (VT1 + VT2), measured in: **a** Cycling efficiency (CE); **b** Gross efficiency (GE); **c** Lactate; **d** Total time until exhaustion. Data are provided as mean and error bars as 95% confidence intervals. There were no significant differences between both experimental conditions BJ vs. PL

The CE, GE and VT2 time trial data are shown in Fig. 2. As occurred for the cardioventilatory variables, there were no significant interaction (supplement*intensity) effects on CE, GE or VT2 time trial (*p* > 0.05). Neither was a significant supplement effect produced on any of the variables (*p* > 0.05). However, as expected, significant intensity effects were produced on CE (F_(2, 22)_ = 12.824, *p* < 0.001, η_p_^2^ = 0.538, SP = 0.992), GE (F_(2, 22)_ = 6.495, *p* < 0.001, η_p_^2^ = 0.733, SP = 1), lactate (F_(2, 18)_ = 24.743, *p* < 0.001, η_p_^2^ = 0.733, SP = 1), and on VT2 time trial (F_(2, 20)_ = 95.114, *p* < 0.001, η_p_^2^ = 0.905, SP = 1).

Although no significant differences (*p >* 0.05) were found between BJ and PL in VT2 time trial and VT1 + VT2 (Fig. 1d), BJ supplementation lead to a shorter VT2 time trial (BJ: 15 min 33 s, PL: 14 min 42 s).

### Energy expenditure, carbohydrate oxidation, fat oxidation and RPE

**Fig. 3:**
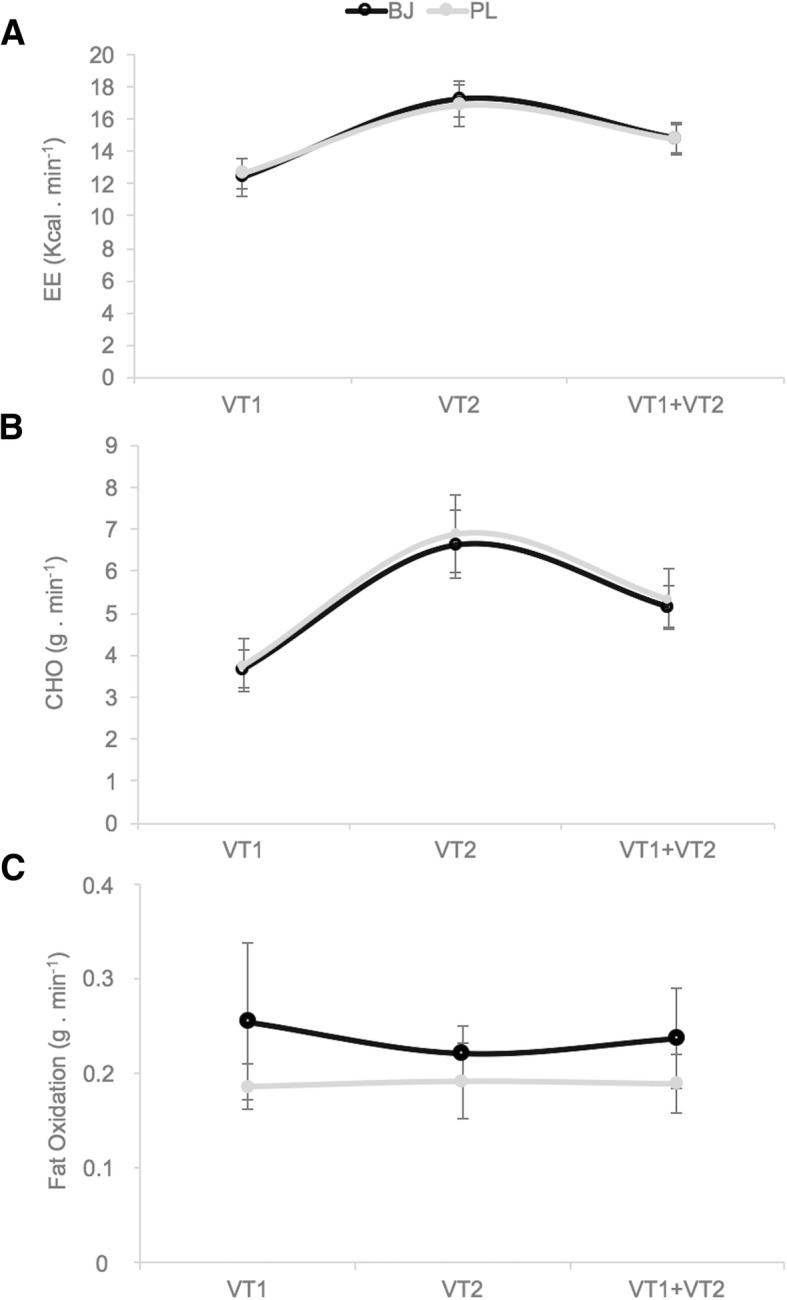
Differences between beetroot juice (BJ) and placebo (PL) at first ventilatory threshold (VT1), second ventilatory threshold (VT2) and in the time to completion (VT1 + VT2), measured as: **a** Energy expenditure (EE); **b** Carbohydrate oxidation (CHO); **c** Fat oxidation. Data are provided as mean and error bars as 95% confidence intervals. There were no significant differences between both experimental conditions BJ vs. PL

Data related to energy expenditure, carbohydrate oxidation and fat oxidation are shown in Fig. 3. No significant interaction or supplement effects on any variable were produced (*p >* 0.05). Significant intensity effects were observed on carbohydrate oxidation (F_(2, 22)_ = 81.339, *p* < 0.001, η_p_^2^ = 0.881, SP = 1), and energy expenditure (F_(2, 20)_ = 91.043, *p* < 0.001, η_p_^2^ = 0.901, SP = 1). No significant intensity effect was detected on fat oxidation (*p >* 0.05). No significant interaction or supplement effects were produced on RPE (*p >* 0.05).

## Discussion

As far as we know, this is the first study to examine the possible effects of acute BJ supplementation on a constant workload cycloergometry exercise conducted at VT1 + VT2 time trial in well-trained endurance triathletes. As VT seems to be the most accurate predictor of endurance performance, especially in cycling [31], this study was designed to test the efficacy of BJ at improving performance during aerobic energy metabolism (VT1) and during the transition from aerobic to anaerobic metabolism (VT2).

Contrary to our working hypothesis, acute BJ supplementation was not observed to improve cardioventilatory responses, mechanical exercise economy/efficiency, slow component, use of energy substrates or performance in these athletes. Even though national athletes were less trained than international athletes, no positive effect of BJ supplementation was observed in both international and national athletes.

Our VT1 data confirm the results of other studies [50, 51] in which neither were improvements observed in cardioventilatory responses to low-moderate intensity submaximal exercise after supplementation with NO_3_^−^. Cristensen et al. (2013) [20] reported no GE increase after the intake of 0.5 L of BJ over 6-day periods (0.5 g nitrate per day). Their test protocol involved different work types including work and rest periods in elite cyclists. In another study, Bescos et al. (2011) [50] also detected no GE improvements in well-trained cyclists and triathletes in response to acute sodium nitrate supplementation (10 mg · kg^− 1^ dissolved in 250 mL of water), in a test in which there was a single transition at different intensities and with a limited rest period. In contrast, others have shown increases in GE [51] and reductions in pulmonary VO_2_ and O_2_ cost in submaximal low-moderate intensity exercise in healthy moderately- and well-trained athletes following the intake of BJ [14] (0.5 L for 6 days, 5.5 mmol per day of NO_3_^−^) and sodium nitrate (0.1 mmol kg^− 1^ bodyweight day^− 1^) [51] using different supplementation protocols and cycle ergometry as the assessment test. There is no consensus on the appropriate dose in well-trained athletes at low-moderate exercise intensity.

Previous studies have demonstrated that higher BJ supplementation dose (~ 8.4 mmol and ~ 16.8 mmol of NO_3_^−^) caused a greater reduction in systolic blood pressure and mean arterial pressure at moderate exercise intensity than lower doses (~ 4.2 mmol of NO_3_^−^) in healthy adults [37]. In this study, VO_2_ steady-state of moderate exercise intensity was reduced significantly after ingestion of 16.8 mmol of NO_3_^−^, tended to be lower after intake of 8.4 mmol NO_3_^−^, and was unaffected by 4.2 mmol of NO_3_^−^ [37]. Higher doses of BJ supplementation (2 × 70 mL doses per day, ~ 6.2 mmol of NO_3_^−^ per 70 mL) before and during prolonged moderate-intensity exercise might be necessary to attenuate the progressive rise in VO_2_ and reduce muscle glycogen depletion [52], improving mechanical efficiency during a prolonged constant-load test at VT1 intensity. Further, IOC consensus statement concludes that longer periods (> 3 days) of NO_3_^−^ supplementation could increase sport performance in highly-trained athletes [22].

It is unclear that dose-response relationship exists between acute BJ supplementation and the physiological mechanisms for the reduction in the O_2_ cost and pulmonary O_2_, decrease in VO_2_ slow component and increases in GE during low-moderate exercise intensity. Probably, the dose used in our study was not enough to cause an ergogenic effect in well-trained triathletes.

The type and/or mode of supplementation or the test used do not seem to play as important a role as cardiovascular fitness level when assessing VO_2_, O_2_ cost and GE at low-moderate intensity, as moderately-trained healthy athletes have shown a favorable response to BJ supplementation as opposed to a negative response of well-trained athletes, regardless of the test or supplementation protocol (acute or chronic). Subjects with a lower fitness level may be more susceptible to BJ effects regardless of whether the BJ supplementation is acute or chronic. In effect, the literature suggests some interaction between training state and the ergogenic effects of NO_3_^−^ supplements [53], though the physiological mechanisms induced remain unclear and are likely related to adaptations achieved in response to endurance training [54]. It is known that the most skilled individuals feature better vascular control, characterized by a greater activity and presence of the enzyme endothelial nitric oxide synthase (eNOS), responsible for endogenous NO production [55]. Thus, any increase in eNOS activity could reduce the availability of NO derived from nitrates, consequently diminishing the possible effects of BJ. This rationale could explain, at least in part, the results obtained here for VT1 as this is a low-moderate intensity of exercise after which a first evident shift is produced in ventilation and in blood lactate concentrations and above which anaerobic energy metabolism is partly involved [56]. Lactate concentrations in our athletes at VT1 were lower than 2 mmol. L^− 1^ indicating a predominantly aerobic state. During this metabolic stage, such intensity of exercise may be maintained over a long period of time without marked changes in blood lactate concentrations [57, 58]. Hence, it is less likely that a trained athlete will experience low muscle oxygenation increasing muscle acidosis and generating nitrate reduction at a given work rate [30]. We suspect there was no tangible effect of BJ, as more trained subjects could show reduced O_2_ uptake due to a decrease in the aerobic energy required or in the muscular energy used in moderate exercise efforts.

The reduction in VO_2_, attributed to reduced ATP resynthesis through oxidative phosphorylation, was not offset by elevated glycolytic ATP provision [14], as indicated by the similar blood lactate concentrations observed in the groups BJ and PL. However, as argued by Bailey et al. (2009) [14], in less trained subjects, a beneficial effect of NO_3_^−^ is produced reflected by increased muscle oxygenation indices and total hemoglobin levels during moderate exercise. The increased blood volume observed in the vastus lateralis muscle after BJ intake is presumably a consequence of improved muscular vasodilation resulting from the increased production of NO from NO_2_^−^.

Compared to VT1, physiological and efficiency (intensity effect) changes were observed here in VT2. Studies have shown that BJ enhances high-intensity endurance exercise performance in moderately-trained subjects [14, 59] while its effects are not so clear in well-trained subjects. In a study conducted in elite cyclists [20], the time taken to complete a time trial failed to vary significantly between individuals given BJ or PL. This is similar to the effects on VT2 observed in the present study (400 kcal-time trial 18:20; VT2 time trial 15:33 min:s, respectively), with comparable levels of power reached (290.0 ± 43.0 W vs 282.1 ± 37.9 W respectively). Thus, it could be that the intensities set in both tests (preload vs. VT1 and 400 kcal-time trial vs. VT2 time trial) gave rise to an aerobic metabolism and transition to an anaerobic energy pathway. In statistical terms, BJ showed no endurance performance-enhancing effect in both studies (0.8% and 5.7% in our study). However, significant performance improvements in response to BJ have been observed in well-trained cyclists and triathletes of 1.2% [17] and in rowers (− 1.6 ± 1.6 s) [23], along with an increase, though not significant, of ~ 2% in trained cyclists and athletes [50], and a beneficial response in some elite athletes [60]. Collectively, these findings point to a possible ergogenic effect of BJ on the cardiorespiratory performance of highly-trained endurance sport athletes. It should be considered that to increase the possibility of winning, a high-level endurance sport athlete needs to achieve a gain in total time of at least 0.6% [61]. For example, the variance between twelfth and first place in the 10,000 m men’s running final at the 2012 London Olympics was only 0.66% [62]. Such a slight biological improvement induced by BJ supplementation (not statistically significant), together with intrinsic and extrinsic motivational factors, could be determining factors for success in high-level athletes and this impact may have been detectable in a larger sample size. It would be logical to assume that BJ supplementation could at least partly influence cardiorespiratory performance especially when small improvements in endurance tests can be particularly meaningful. Because these changes in performance are so small, it would be noteworthy to evaluate the differences between physiological and motivational factors produced by BJ supplementation.

Currently, the scientific literature lacks data on the effects of nitrates on high-intensity exercise [53] and the assumption gains importance that BJ supplementation could improve the capacity to cope with fatigue in situations of transition from an aerobic to anaerobic energy metabolism, despite a poor understanding of the physiological etiology involved in well-trained athletes. This is especially true as the slight, yet interesting, increase in VT2 time trial took place in the absence of a beneficial impact of BJ on the cardioventilatory response, exercise economy/efficiency, slow component, use of substrates and blood lactate concentrations. Maybe, an increase in the BJ supplementation dose would have been a factor key to detect improvements in the variables analyzed in our study. Previous findings have shown that higher BJ supplementation dose (~ 8.4 mmol and ~ 16.8 mmol of NO_3_^−^) improves the time-to-task failure when is compared with a 4.2 mmol dose in young healthy adults [37]. Effectively, in well-trained subjects it would be necessary to intake larger NO_3_^−^ doses (140 mL, ~ 8.4 mmol, 550 mg of NO_3_^−^) [23]. A normal dose (70 mL) is unlikely to trigger an ergogenic effect. Higher dose (~ 16.8 mmol of NO_3_^−^) raises the plasma NO_2_^−^ levels to a greater extent than ~ 8.4 mmol of NO_3_^−^, however, no added performance gains are produced [23]. Prolonged periods of BJ supplementation longer than 3 days could increase sports performance [24, 25] and could be used as an alternative supplementation strategy for well-trained athletes.

Furthermore, whole vegetables have been demonstrated to provide important health benefits whereas NO_3_^−^ from other sources could lead to adverse effects on health [63]. Because NO_3_^−^ consumed in the form of vegetables have been shown to improve running performance in healthy adults [63], it is tempting to speculate that supplementation strategy based on whole beetroot could be an interesting choice for well-trained competitive athletes while preserving their health. More studies analysing the effects of whole vegetables intake on endurance performance in well-trained athletes are necessary to substantiate such claims.

As a final remark, changes in exercise intensity from VT1 to VT2 involve variations in VO_2_, leading to the use of different substrates. Carbohydrates are more efficient as energy substrates than fatty acids. In other words, if more carbohydrates are used as substrate this gives rise to lower oxygen absorption at a given work velocity [51]. Calculations of GE include possible RER changes and, therefore, take into account substrate use. Neither did BJ seem to induce more efficient substrate use as reflected by our data for GE, RER, and consumption of energy, carbohydrates and fats in both experimental groups. Further, our GE calculations were targeted at assessing the effects of blood alkalization on gradual losses in muscle efficiency as the best indicator of the so-called slow component phenomenon [64]. With this protocol inducing a change in metabolism from VT1 to VT2, we sought to examine the effects of BJ after promoting a change in VO_2_ kinetics (slow component). This change is similar to that observed after an initial bout of high-intensity exercise, giving rise to increased muscle O_2_ release, increased oxidative metabolic enzyme activity, carbon substrate availability, and abnormal motor unit recruitment patterns [65, 66]. It is not clear in the scientific literature whether any of these physiological mechanisms could reduce the slow component in response to BJ supplementation in healthy moderately-trained subjects [14, 67]. In a recent study, Tan et al. (2018) [52] demonstrated that BJ supplementation mitigated the progressive rise in VO_2_ over time before and during prolonged moderate-intensity exercise although did not enhance subsequent time trial performance. Interestingly, it was observed that this decrease in VO_2_ had no impact on time trial performance, which could indicate that supplementation with BJ does not sufficiently reduce muscle glycogen depletion at moderate intensity for decreasing fatigue during cycling time-trial. More research is needed to analyze the BJ supplementation effect on VO_2_ kinetics during endurance tests over two hours.

There are some limitations in this study which should be considered. Previous research indicates that the plasma nitrite of the participants should increase to show an ergogenic effect, however, nitrite and nitrate concentrations in plasma were not measured in our study. It seems that doses close to or greater than 8.4 mmol are more adequate to determine the positive effects of BJ supplementation on endurance performance in well-trained triathletes [23].

The small sample size in this study should be taken into account when drawing conclusions from the data. Minimal changes in endurance performance are usually observed in well-trained triathletes, therefore, large sample sizes should be required to detect significant changes produced by BJ supplementation on cardioventilatory performance.

Although there are several studies that have carried out a similar washout period, it is possible that the washout period established in our study was not sufficient, which could influence the final results.

## Conclusions

Our findings do not support an improvement in the variables examined here produced in response to acute BJ supplementation. We have yet to elucidate the possible ergogenic effects of BJ in highly trained athletes. However, the slight (not significant) modifications observed in performance variables such as test duration or maintaining work intensity at a given load in several studies prompts numerous questions as the mechanical and physiological mechanisms analyzed so far do not support these improvements and remain poorly understood.

Our outcomes suggest a need to analyze individual positive responses to this form of supplementation in well-trained athletes.

## Abbreviations

ANOVA: Analysis of variance
BMI: Body mass index
CE: Cycling efficiency
CI: Confidence intervals
GE: Gross mechanical efficiency
NO: Nitric oxid
NO_2_^−^: Nitrite
NO_3_^−^: Nitrate
PetCO_2_: End-tidal partial pressure of carbon dioxide
PetO_2_: End-tidal partial pressure of oxygen
PL: Placebo
RER: Respiratory exchange ratio
RPE: Rating of perceived exertion
rpm: Revolutions per minute
SP: Statistical power
VE: Minute ventilation
VE·VCO_2_^− 1^: Ventilatory equivalent for carbon dioxide
VE·VO_2_^− 1^: Ventilatory equivalent for oxygen
VO_2_: Oxygen uptake
VO_2_max: Maximum oxygen uptake
VT1: First ventilatory threshold
VT2: Second ventilatory threshold
W: Watt
η_p_^2^: Partial eta-squared
